# Deciphering the dynamic code: DNA recognition by transcription factors in the ever-changing genome

**DOI:** 10.1080/21541264.2024.2379161

**Published:** 2024-07-20

**Authors:** Yumi Minyi Yao, Irina Miodownik, Michael P. O’Hagan, Muhammad Jbara, Ariel Afek

**Affiliations:** a Department of Chemical and Structural Biology, Weizmann Institute of Science, Rehovot, Israel; b School of Chemistry, Raymond and Beverly Sackler Faculty of Exact Sciences, Tel Aviv University, Tel Aviv, Israel

**Keywords:** DNA, transcription factor, repair enzyme, protein-DNA interaction, epigenetic modification, DNA damage, DNA mechanics

## Abstract

Transcription factors (TFs) intricately navigate the vast genomic landscape to locate and bind specific DNA sequences for the regulation of gene expression programs. These interactions occur within a dynamic cellular environment, where both DNA and TF proteins experience continual chemical and structural perturbations, including epigenetic modifications, DNA damage, mechanical stress, and post-translational modifications (PTMs). While many of these factors impact TF-DNA binding interactions, understanding their effects remains challenging and incomplete. This review explores the existing literature on these dynamic changes and their potential impact on TF-DNA interactions.

## Introduction

**Figure 1.**
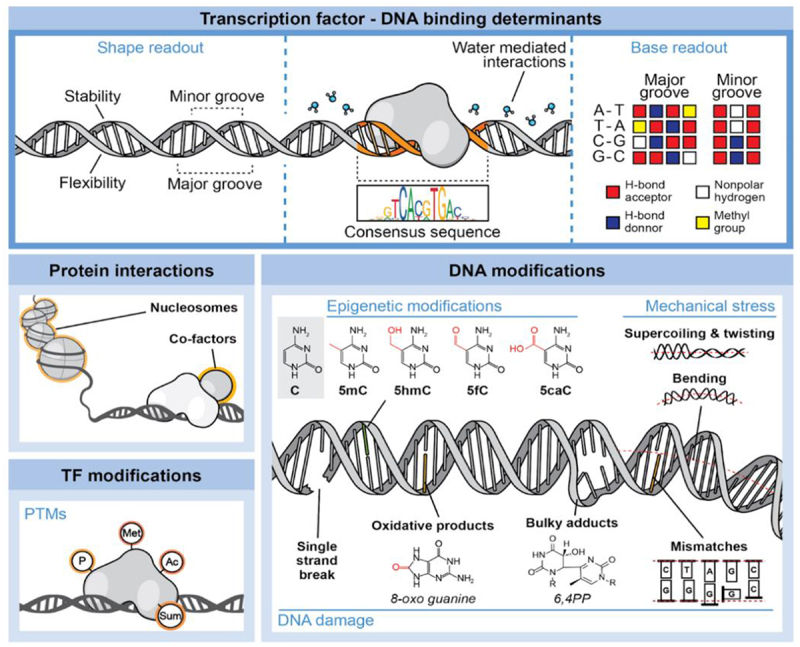
Overview of the primary (shape/base readout), auxiliary (protein/co-factor interactions) and dynamic (epigenetic, damage, mechanical and post-translational modifications) components that determining TF/DNA recognition.

Transcription factors (TFs) bind specific genomic DNA sequences to control complex gene expression programs [1], and misregulation of TF function is observed in many diseases such as cancer and neurodegeneration [2,3]. Determining the genomic rules that govern TF-DNA recognition is therefore a vital step toward understanding the role this process plays in health and disease. Over the past five decades, researchers have extensively investigated the mechanisms governing the sequence-specific recognition of DNA by TFs, summarized in Figure 1, seeking to unveil the key determinants that govern this process [4–8]. These factors include direct interactions with the chemical groups of DNA bases, indirect structural effects, water-mediated interactions, as well as additional weak interactions with the sequences surrounding the target sites [9–13]. Alongside these primary components, auxiliary components play a pivotal role in modulating TF-DNA binding interactions within cells. These include other DNA-binding proteins, co-factors, and histone proteins that envelop DNA. While much research has investigated the roles of these primary and auxiliary components in governing TF-DNA binding [14–18], we still do not fully understand how the continuous chemical and mechanical changes DNA and DNA-binding proteins experience in the dynamic cellular environment affect these components of TF-DNA recognition.

Dynamic alterations include epigenetic DNA modifications, DNA damage, protein post-translational modifications (PTMs), and structural deformations [19–22]. Despite growing insight into the significance of these alterations, investigating their full impact poses challenges – and a comprehensive understanding of their influence on TF-DNA recognition is yet to be achieved. In this review, we consider the potential influence of these dynamic changes in regulating TF-DNA interactions, drawing on recent literature investigating these effects and identifying the key areas for the development of the field.

### Beyond the four-letter alphabet

The fundamental units of DNA, responsible for encoding genetic information, consist of the four nucleotides Adenine (A), Thymine (T), Guanine (G), and Cytosine (C). These nucleotides form complementary base pairs (bp) within two strands, giving rise to the canonical double-helical structure. However, the four-letter DNA alphabet and the canonical structure only offer a partial depiction of the nucleobase diversity and structures present in the genome. This diversity arises from the dynamic nature of the genome, which undergoes constant chemical modifications, such as methylation, oxidation, and deamination of nucleobases, as well as mechanical stresses, such as DNA bending and twisting during replication and transcription. These alterations in DNA chemistry and its 3D structure are highly likely to affect its interactions with TFs.

### Transcription factor target recognition in a dynamic genome

**Figure 2.**
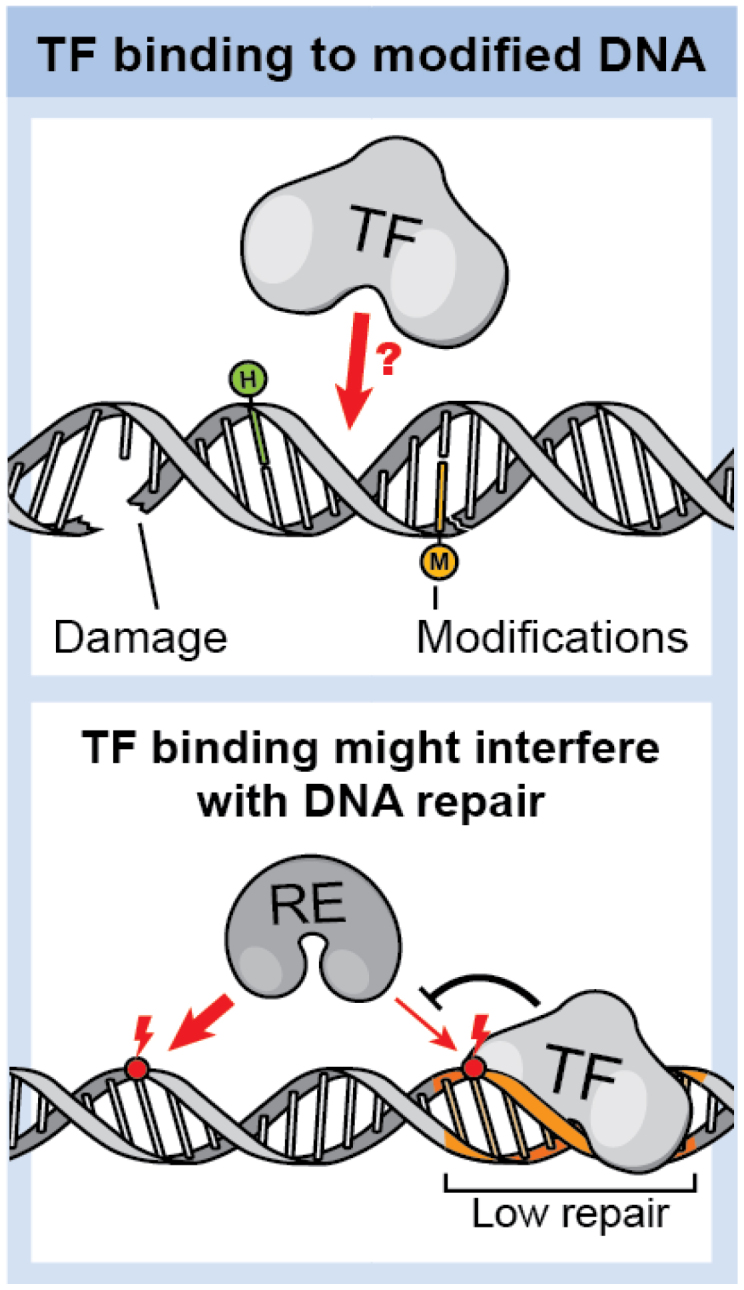
TF binding to modified DNA. Upper panel: DNA damage/modification may influence TF binding. Lower panel: competitive binding of TFs at damaged sites may impair repair enzyme efficacy.

The DNA-binding domain of a TF can identify its target site by sensing the physicochemical characteristics at the edges of base pairs. Each nucleotide displays a unique chemical signature, allowing TFs to selectively bind to specific strings of nucleotides, commonly referred to as sequence motifs. This type of sequence recognition is known as “direct readout” or “base readout”. The chemical signatures, encompassing hydrogen bond (H-bond) acceptors and donors, methyl groups, and nonpolar C-H bonds, are accessible on the surfaces of the DNA major and minor grooves. These accessible chemical groups facilitate the formation of various contacts with TF residues, including H-bonds, water-mediated bonds, and hydrophobic interactions [8]. In the dynamic genome, specific enzymes, such as DNA methyltransferases, and distinct chemical entities, such as reactive oxygen species, engage in reactions with DNA – leading to the modification of nucleobases or formation of strand breaks. These modifications alter the chemical signature of nucleotides and may significantly influence the “direct readout” of TFs (Figure 2, upper panel).

In addition to direct readout, the identities of nucleotides within a sequence can impact binding, even when the chemical groups of specific bases do not directly interact with the residues of a TF. This is because the nucleotide sequence influences additional elements – such as DNA stability, flexibility, and groove width – known to play a role in the binding mechanism. This type of indirect sequence-dependent recognition is widely referred to as “indirect readout” or “structural readout” [8,23–25]. The chemical modifications occurring in the dynamic genome can alter these local structural characteristics of DNA. The nature and severity of these structural changes depend on the type of the modification and may lead to different alignments of other chemical groups that are capable of forming interactions with the TF. Additionally, various processes in the genome – including replication, repair, and transcription – may induce mechanical stress on the DNA, significantly altering its canonical structure. All of these changes may impact the “structural readout” of various TFs in the genome.

Integrating this understanding with the roles of cofactors, nucleosomes, and protein–protein interactions further complexifies the mechanism of TF-DNA recognition. Cofactors and nucleosomes modify the chromatin environment, either facilitating or hindering TF access to DNA [26–28], while interactions among TFs can synergistically enhance or competitively inhibit their collective binding affinity and specificity [29–31]. Beyond this, the spatial arrangement of nucleosomes itself can dictate the accessibility of specific genomic regions, acting as a dynamic scaffold that either exposes or conceals binding sites from TFs [27,32–34]. This nuanced interplay significantly impacts gene expression patterns, with cofactors acting as modulators that can recruit or repel TFs from their target sites by altering the physical state of chromatin. Consequently, the orchestration of these elements – cofactors, nucleosomes, and TF interactions – creates a regulatory network that finely tunes gene expression. In the dynamic genome, chemical changes in DNA can modify the binding of certain TFs, subsequently influencing the binding of other partnering TFs. These alterations may also change the positioning of nucleosomes, contributing to variations in accessibility at genomic locations where TFs may bind. This chain of interactions, initiated by chemical and structural changes in the DNA, can have a secondary but significant impact on TF-DNA recognition in cells.

In addition to the influence that TF-DNA interactions with modified DNA can have on cellular transcription activity, they may also impact cellular repair efficiencies and contribute to the formation of mutations – specifically in cases of DNA damage.

When DNA damages occur, different repair pathways are activated in the cell to remove the DNA lesions [35]. However, repair is not uniform throughout the genome, resulting in higher mutation rates at certain locations [36]. Recent evidence strongly suggests that proteins binding to damaged DNA can disrupt the functionality of the repair machinery, leading to an elevated frequency of DNA mutations, particularly at the sites where the TFs are bound [37,38]. Therefore, to gain a deeper understanding of mutation formation, it is essential to understand how TFs bind to various unrepaired damages, ultimately contributing to these mutations (Figure 2, lower panel).

In the following sections, we will discuss different modifications and how they may affect TF-DNA interactions.

## DNA epigenetic modifications

DNA epigenetic modifications play a pivotal role in regulating gene expression and orchestrating various cellular processes in mammalian organisms. These chemical modifications of DNA bases are widely prevalent and tightly regulated, exerting profound effects on fundamental cellular mechanisms such as X chromosome inactivation, the silencing of transposable elements, and genomic imprinting [39].

**Figure 3.**
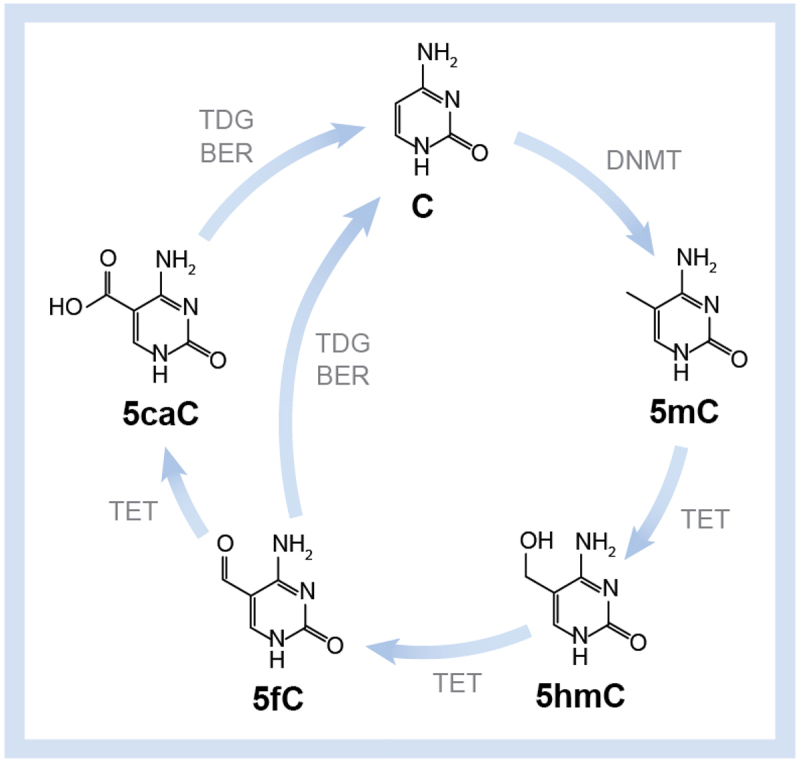
Cycle of enzyme-mediated cytosine methylation/demethylation. Initial methylation of C to 5mC by DNMT is followed by oxidation to 5hmC, 5fC and 5caC by TET. 5fC and 5caC are restored to unmodified C by TDG through the base excision repair (BER) pathway.

5-Methylcytosine (5mC), often referred to as the fifth base, represents the most common epigenetic modification, occurring in approximately 80% of genomic CpG dinucleotides (CpGs) in somatic cells [40]. This modification arises upon methylation of cytosine by the DNA methyltransferase (DNMT) family of enzymes [39,41]. Another enzyme family, the Ten Eleven Translocases (TET), oxidizes 5mC to 5-hydroxymethylcytosine (5hmC), 5-formyl cytosine (5fC), and 5-carboxyl cytosine (5caC), thus expanding the diversity of epigenetic markers [42]. In turn, 5fC and 5caC can be restored to unmethylated cytosine through the Base Excision Repair (BER) pathway. This cycle of enzyme-mediated cytosine methylation/demethylation is depicted in Figure 3. Each of the base modifications in this pathway is known to influence gene expression [43–45] and also have the potential to affect TF binding. In this section, we will briefly discuss current knowledge relevant to the effect of DNA epigenetic modifications on the binding of TFs to DNA.

### 5-methylcytosine (5mC)

The impact of cytosine methylation on TF binding has been extensively studied and is the focus of several reviews [46,47]. 5mC can induce changes in the chromatin structure that decrease TF accessibility to DNA [48], which causes transcriptional silencing of certain genomic regions. It can also directly alter the physical interactions of TFs with DNA; the methyl group may either introduce additional steric hindrance in the protein-binding pocket, disrupting interactions present in the case of the unmethylated analog, or confer additional stability through the formation of new hydrophobic interactions. The direct influence might be particularly of interest *in vivo*, as observations show that certain promoters can be simultaneously active and methylated [49], and within distal enhancers, methylation levels can reach significantly high levels [50].

Beyond these direct effects, a growing body of evidence suggests that 5mC can also reshape various structural properties known to influence TF binding. Structural analysis revealed that CpG methylation narrows the minor groove width and increases the DNA roll [51]. In the same study, it was demonstrated that high-resolution DNase I cleavage profiles can provide detailed information about the structural changes caused by methylation as the intrinsic rate of cleavage closely tracks the width of the minor groove. Recent studies have also demonstrated that methylation alters the tendency of different DNA sequences to bend [52].

Specific cases of the direct impact of methylation on TF binding have been reported for decades (e.g [53–59]). For example, CTCF was one of the first proteins where binding affinity was linked to 5mC regulation, specifically in relation to imprinted genes [58], and the negative impact of methylation on NRF1 binding has been studied *in vivo* [59]. However, the recent introduction of several *in vitro* and computational high throughput methods has allowed a more systematic exploration of interactions between TFs and methylated CpG. These methods, such as methylated protein-binding microarrays [60], methyl-spec-seq [61], bisulfite-SELEX [62], epi-SELEX [63], methyl-DNAshape [64] and others, have facilitated the determination of binding preferences of hundreds of TFs to sites containing methylated CpG, greatly increasing our understanding of the global sequence preferences.

Binding of many TFs from the BZIP, BHLH, ETS and RUNT families is generally found to be inhibited by CpG methylation (for example, ZFP57, ATF4/7, CEBPD/G, ETS1 and BATF1 [61–63]). The detrimental effect of the methyl group on binding is proposed to arise from increased steric hindrance and certain changes in the DNA shape, such as in roll and propeller twist, which in turn affect base pairing and stacking in the protein-binding site [64].

**Figure 4.**
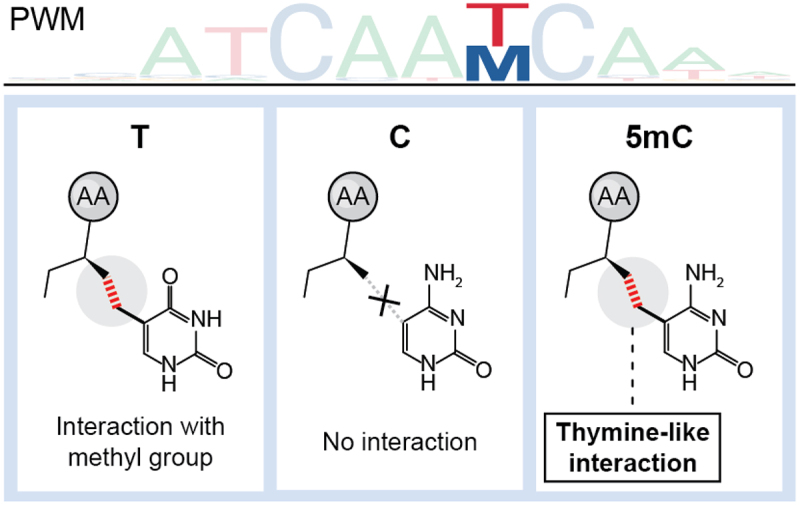
Thymine mimicry by 5mC. Upper panel: schematic position weight matrix (PWM) indicating similar binding preference for thymine (T) and 5mC(M). Lower panel: mimicking of hydrophobic interaction between thymine and protein residue is possible by 5mC but not unmethylated C.

On the other hand, the binding of many other TF families, including homeodomain (e.g., HoxA9, HoxB13 and HoxC11), POU (e.g., Oct4) and certain zinc fingers (e.g., UHRF1 and KLF2/4/5), are positively influenced by methylation. Remarkably, an Oct4 ChIP-seq experiment in TET1–3 knockout cells revealed an entirely new consensus-binding site that contains methylated CpG [62]. While there is no universal mechanism explaining the recognition of methylated CpG, for certain TFs, the recognition can be explained through a concept known as “thymine mimicry” (Figure 4). In these cases, the preferred interaction with non-methylated DNA is mainly driven by an amino acid interaction with the methyl group of thymine. Given that 5mC bears a methyl group in the same position as thymine, it can “mimic” this interaction pattern and form an equally strong interaction with the protein [63].

**Figure 5.**
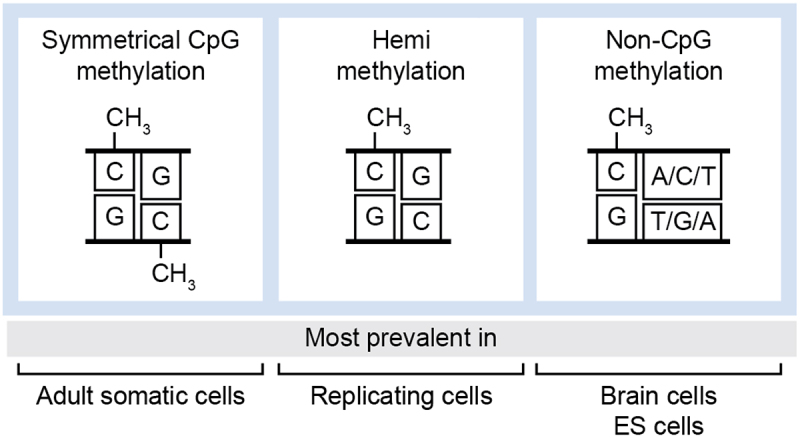
Different cytosine methylation patterns. Left panel: symmetrical CpG methylation within CpG dinucleotides and in both DNA strands. Central panel: hemi methylation within CpG dinucleotides, in only one of the DNA strands. Right panel: Non-CpG methylation within CpA, CpC or CpT dinucleotides.

Whilst symmetrical methylation of CpG (both strands) is most common (Figure 5, left panel), in every replication cycle, hemi-methylation (methylation of only one strand) is also observed (Figure 5, middle panel). UHRF1, a DNA-binding protein that participates in the methylation maintenance process, is a well-known case that exhibits a preference for hemi-methylated DNA [65,66]. Whilst this suggests a regulatory role for hemi-methylation, the global influence of hemi-methylated DNA on TF binding remains underexplored. It is important to note that while 5mC occurs most frequently within the context of CpG dinucleotides, non-CpG methylation is also observed (Figure 5, right panel). Remarkably, in certain cell types, such as brain cells and embryonic stem cells, non-CpG methylation may constitute around 25% of the total cellular methylome [67]. Although the overall impact of non-CpG methylation on TF binding remains incompletely understood, studies demonstrating a negative correlation between non-CpG methylation and gene expression [67], as well as associations between abnormal levels of non-CpG methylation with certain cancer types [68], hint at a potential regulatory role for non-CpG methylation in determining DNA recognition by TFs.

### 5-hydroxymethylcytosine (5hmC)

5-hydroxymethylcytosine is the first oxidation product of 5mC in which the methyl group is converted to a primary alcohol. Whilst this modification is comparatively rare in comparison to 5mC, there is emerging evidence supporting its physiological relevance, including in changing transcription factor binding, regulation of gene expression [43], and maintaining the undifferentiated state of embryonic stem cells [69].

For instance, certain proteins such as UHRF2 bind both fully hydroxymethylated and hemi hydroxymethylated DNA with higher affinity than the methylated or unmethylated variants [70]. Structural studies revealed that the recognition of the modification in this case occurs through a hydrophobic pocket that interacts with the pyrimidine ring of the flipped out 5hmC, and a hydrogen bond between the hydroxyl group from the modified cytosine to a carbonyl in the protein [70]. In contrast, other transcription factors, such as MAX, WT1 and EGR1, have a markedly decreased affinity for hydroxymethylated DNA [44,45]. Several studies have shown that 5hmC, as well as 5mC, stabilizes the DNA double helix [71,72], and increases flexibility [73]. As double helix stability is known to be higher at transcription factor-binding sites [74], and higher flexibility allows the DNA to adopt the correct shapes for interaction with TFs, hydroxymethylated DNA might generally favor certain TF binding. Additionally, the hydroxyl group in 5hmC possesses higher polarity compared to the methyl group. While this increased polarity may potentially favor the formation of new interactions with polar amino acids, the steric hindrance from the hydroxyl group and the resulting polarity changes may also reduce binding, as observed in several systems [44,45]. However, to date, the impact of 5hmC has been explored only for a limited number of TFs, and its full implications remain far from understood. We anticipate that with the advancement of genome-wide methods for accurately mapping 5hmC patterns across the genome [75,76], coupled with the development of novel high-throughput approaches for TF-DNA binding studies, this knowledge gap will gradually diminish in the coming years.

### 5-carboxyl cytosine (5caC) and 5-formyl cytosine (5fC)

Much less is known about the effects of 5caC and 5fC on binding of transcription factors, as these modifications were merely considered as demethylation intermediates until recently. However, their potential regulatory roles are gradually gaining the attention of the research community, as evidence accumulates that these modifications may have physiological implications for transcription factor binding. Some observations suggest that 5fC changes the DNA conformation and leads to DNA unwinding [71], and reports also show that it increases DNA flexibility [73]. Because the binding mechanism of several TFs involves changes in the DNA structure, changes in these parameters are likely to alter their binding preferences. Indeed, a few examples of the impact of 5caC and 5fC on TF binding have been discovered to date. For instance, the transcriptional regulator MAX exhibits similar affinity for a site containing 5caC as for an unmethylated site, while sites containing 5fC, 5mC and 5hmC displayed reduced affinity [44].

Egr1 and WT1 are also known to discriminate between the demethylation intermediates of cytosine. 5mC and C bind with highest affinity, whilst affinity for 5hmC and 5fC is lower. In the case of Egr1, affinity for 5caC is also lower than 5mC and C, whilst WT1 does not exhibit this preference [45].

Notably, 5fC and 5caC are actively recognized by cellular DNA repair machinery, particularly the TDG repair enzyme, during the demethylation process. Thus, the differential binding sensitivity of TFs to these modifications might indicate a mechanism of transcriptional regulation through the TET mediated pathway [44]. For example, the affinity of MAX increases at each oxidation step in the demethylation mechanism, suggesting expression levels of its targets might increase gradually with demethylation.

Strong binding of TFs to 5caC and 5fC may also influence TDG binding. TFs might interact with TDG and assist in recruiting this enzyme to the correct sites, thereby increasing the efficiency of the repair process. On the other hand, TF occupancy might interfere with the repair process and yield the opposite effect. We anticipate that further research on this topic will emerge in the near future, shedding light on these questions and providing clearer answers.

To summarize, especially in recent years, much progress has been made in understanding the implications of DNA epigenetic modifications on DNA binding and gene regulation. Nowadays, the binding preferences for methylated or unmethylated CpG sites are available for hundreds of transcription factors. However, there is still a gap in our knowledge regarding how other types of methylation, such as non-CpG methylation and hemi-methylation, might regulate transcription factor binding. Similarly, our understanding of the full impact of rarer epigenetic modifications (i.e., 5hmC, 5fC, and 5caC) on transcription factor binding remains incomplete.

## Dna damages

In a similar way to epigenetic modifications, DNA damage involves chemical alterations to the DNA molecule. However, in contrast to most epigenetic modifications that are undertaken by enzymes in a controlled manner and typically cause relatively minor changes to the structure of the DNA, the impact of DNA damage is often much more severe and commonly deleterious. Estimates suggest that each cell in the human body undergoes thousands of DNA damages daily [77]. Damage formation is attributed to various internal factors, including reactive oxidative species (ROS), S-adenosyl-L-methionine (SAM), polymerase incorporation errors [78], and others. Additionally, these damages can arise from external sources such as UV light, γ-radiation, and smoking [79]. The generation of damage is often correlated with mutation formation, and represents a keystep in initiating carcinogenesis [80]. Recent studies have revealed that DNA damage can persist beyond a single-cell cycle, segregating unrepaired DNA into daughter cells for multiple generations [81]. Additionally, investigations have indicated that TF binding to sites in the genome can commence immediately after DNA replication, even before nucleosomes reassemble on the DNA [82]. These and other findings suggest that damaged DNA, similar to epigenetically modified DNA, may encounter and interact with TFs in the genome.

The impact of DNA damage on TF binding may result not only from direct interactions of the protein with damaged DNA but also from auxiliary interactions with enzymes designated for repairing the damaged site [83]. Understanding the alterations in TF binding resulting from DNA damage is crucial not only due to the pivotal role of TF binding in gene regulation but also because TFs bound to damaged sites may act as roadblocks to repair enzymes [38]. This interference can potentially impede DNA repair in TF-binding sites and lead to the formation of mutations [37,84–86]. Therefore, although damages have a shorter lifetime than some epigenetic modifications, understanding the interplay between DNA damage and protein–DNA interactions is essential for elucidating the pathways of cellular response to DNA damage and the underlying mechanisms of diseases such as neurodegeneration and cancer.

In this section, we will discuss the effects of significant DNA damages observed in the human genome and their implications for transcription factor binding, encompassing oxidative damages, DNA mismatches, single-strand breaks, and other types of DNA damage.

### Oxidative damage

**Figure 6.**
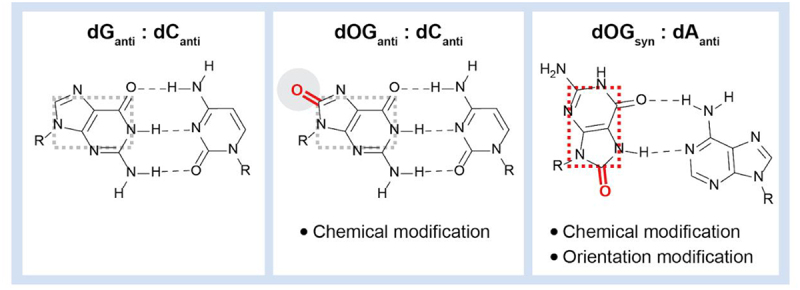
Comparison of base pairing possibilities of G and 8-oxo-G. Left panel: classical G:C base pair (three hydrogen bonds) with both bases in the anti conformation. Middle panel: analogous anti/anti 8-oxo-G:C base pair with the oxo modification oriented in the major groove. Right panel: flipping of 8-oxo-G to syn conformation relieve strain causes mutagenic mispairing with a through formation of two hydrogen bond Hoogsteen base pair.

Reactive oxygen species (ROS) induce substantial cellular stress and deleterious processes, including oxidative damage of DNA [87]. The oxidation of guanine residues into 8-oxo-7,8-dihydroguanine (8-oxodG), alongside other oxidative products [88,89], is particularly common. Indeed, 8-oxo-dG emerges as one of the most prevalent forms of DNA lesions. In normal tissues, 10^3^ 8-oxo-dG lesions are estimated to form per cell per day. However, in cancer cells, this value can reach up to 10^5^ lesions per cell [90,91]. Not only is 8-oxo-dG recognized as a biomarker indicative of oxidative stress [92], but it is also mutagenic and implicated in numerous human pathologies [93]. 8-oxo-dG is generated through the oxidation of guanine at carbon 8 (C8) of the purine base, installing an oxo group at this position with concomitant protonation of the adjacent nitrogen (N7). Following oxidation of a guanine within a canonical dG:dC pair, the anti-conformation and formation of three hydrogen bonds between the bases, as seen in the canonical pair (Figure 6, left panel), can also form in the 8-oxo-dG:dC damaged pair (Figure 6, central panel). However, the addition of the oxo group affects the chemical signature of the major groove, destabilizing the DNA, and potentially leading to differences in transcription factor recognition patterns.

When 8-oxo-dG assumes its usual anti-conformation, the oxygen atom at C8 may encounter steric hindrance with the adjacent phosphate backbone. To alleviate this clash, the base can rotate around its glycosidic bond, adopting the syn conformation. In this syn conformation, a new hydrogen-bonding interface is exposed, enabling the formation of a Hoogsteen base pair with dA (Figure 6, right panel). In this conformation, the 8-oxo-dG lesion is mutagenic, ultimately resulting in a G:C to T:A point mutation, but also has the potential to change both the direct base readout and indirect structural readout by transcription factors.

Indeed, the substitution of guanine with 8-oxo-dG in DNA significantly impairs the binding efficiency of the transcription factor Sp1. The severity of disruption of the binding interaction depends on which position in the binding site is substituted. Such damage-induced perturbation of TF binding can potentially influence gene expression and contribute to age-related diseases and cancer [94]. In vivo findings have indicated that the artificial alteration of 8-oxo-dG within certain promoter regions, including those in the VEGF83, TNFα84, BCL285, and SIRT186 [95–98] genes, can stimulate the transcription of reporter genes [99]. Interestingly, even though oxidative stress leads to the oxidation of the VEGF promoter, it has been observed that decreased SP1 binding to G-rich elements paradoxically enhances transcription [100]. Moreover, in GC-rich Sp1 binding sites, 8-oxo-dG enhances gene transcription in adipose tissue of juvenile mice [101]. Taken together, these studies suggest that 8-oxo-dG plays an important role in the recognition of DNA by the Sp1 protein and consequently in downstream gene regulation.

Other less frequent oxidation products, such as 8,5'(S)-cyclo-2'-deoxyadenosine (cyclo-dAdo) and 8-oxo-7,8-dihydro-2'-deoxyadenosine (8-oxo-dAdo), also influence transcription factor binding in a sequence-specific manner. Introduction of cyclo-dAdo into the target sequence markedly diminishes the binding activity of HSF1, CREB, and NF-kappa B, though HMGA maintains a degree of its binding capability. Conversely, the introduction of 8-oxo-dAdo exhibited a negligible impact on the binding activities of HSF1 and HMGA, especially when compared to DNA that is free of such lesions [102]. However, the impact of these oxidation products on DNA binding proteins within the cellular environment remains largely unexplored, primarily due to the challenge of introducing the damage at specific sites in a controlled manner.

The examples above demonstrate that the introduction of oxidative damage in a transcription factor-binding site can alter TF affinity. However, the degree to which this perturbation impacts gene regulation is still not fully understood. Moreover, sequence-specific rules for the recognition of oxidative damages by TFs have not yet been elicited. We anticipate that recent advances in genome-wide methods for mapping 8-oxo-dG [103], along with improved in vitro high-throughput binding assays, will contribute to such understanding. This will create a new avenue for exploring an additional layer in the study of DNA damage mechanisms in health and disease.

### Single-strand breaks

Single-strand breaks (SSBs) arise through cleavage of the phosphate ester backbone of the DNA biopolymer. SSBs may arise as a result of oxidative stress, but can also be generated as an intermediate or product of many other cellular processes, such as the base-excision repair (BER) pathway, during the formation of DNA replication forks and following abortive ligation events [104]. SSBs are the most common types of DNA lesions occurring in cells, with a frequency of tens of thousands per cell daily [104]. When the single-strand break repair (SSBR) mechanism is defective, unrepaired SSBs can lead to genetic diseases, among which at least six are known and related to neurodegenerative disorders [105]. SSB disrupts the continuous helical structure of DNA, leading to a local distortion of the DNA backbone. This distortion can affect the neighboring base pairs and alter the overall conformation of the DNA molecule in the vicinity of the break [106]. Single-strand breaks might also change the flexibility of DNA [107]. All these structural changes alter the shape of the DNA and therefore have the potential to impact the indirect readout of DNA by TFs.

SSBs are usually accompanied by aberrant 3'- and/or 5' termini, determined by their mechanism of formation [105,108]. For example, an SSB induced by Topoisomerase 1 exhibits a 5’-hydroxyl (5’-OH) terminus following loss of the terminal phosphate [109]. The absence of the phosphate could potentially lead to significant changes in the recognition of proteins, since most DNA-binding proteins form direct interactions with these residues which are important in the stabilization of the protein-DNA complex.

Low-throughput measurements have been conducted to investigate the effect of single-strand breaks on the binding of selected TFs. As an interesting example, the E2 protein (exhibiting a dimeric β-barrel structure) induces a substantial deformation of the DNA as it conforms to the surface of E2 [110]. While, HPV-18 E2/D demonstrates a notably lower affinity to nicked E2BS(AATT) compared to its binding affinity for the corresponding intact oligonucleotide, the introduction of a nick in a DNA target that lacks conformational complementarity to the E2 DNA-binding surface (E2BS(TTAA)) results in a slight enhancement of the interaction affinity [111]. The same was observed for HPV-16 E2/D. This example gives a glimpse into the interplay between DNA flexibility and transcription factor binding. However, it remains largely unknown how single-strand breaks influence the binding of most TFs, and the role of the terminal phosphate residue in each case is particularly unclear. High-throughput platforms and a systematic approach are needed to assist in elucidating the effect of single-strand breaks on TF-DNA recognition and binding.

### Mismatches

DNA mismatches arise when two non-complementary bases align on opposite strands of a DNA duplex, forming non-Watson-Crick base pairs [112]. Various events throughout the cellular life cycle – such as nucleotide misincorporation in DNA replication, genetic differences between recombining helices in genetic recombination, and spontaneous chemical reactions (e.g., nucleotide deamination) [112–117]— frequently give rise to mismatches. While most DNA mismatches formed during replication are promptly repaired, persistent mismatches are a significant source of mutation and have been linked to the development of diverse genetic diseases [118–120]. Mismatches also introduce substantial changes in the DNA helical structure and stability [116,121–126]. Given that base composition and the local DNA shape significantly contribute to the specificity and affinity of protein binding [8,127], mismatches are likely to exert a substantial influence on the recognition of DNA by TFs.

**Figure 7.**
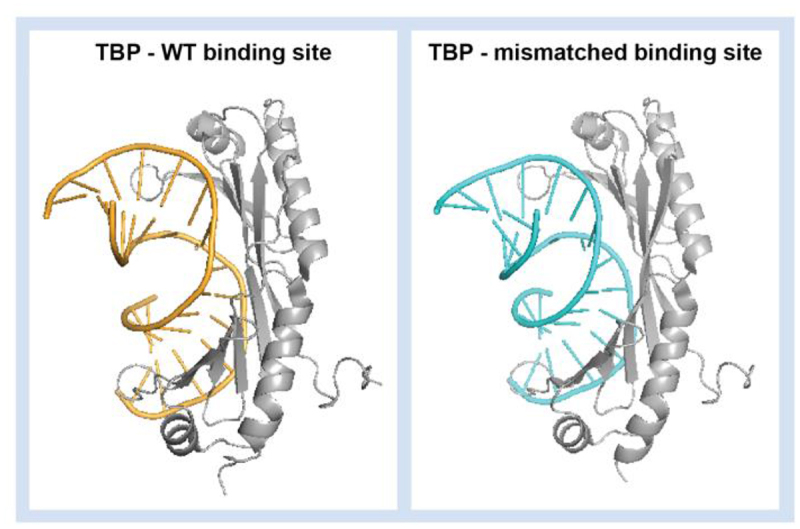
Crystal structure of A. thaliana TBP bound to (left) wild-type DNA binding site (PDB: 1QNE) and (right) binding site with a C-C mismatch (PDB: 6UEP). Preservation of DNA/TBP interactions in the two structures suggests C-C mismatch improves affinity by pre-organization of the DNA into the active binding conformation.

Recent high-throughput studies have provided unprecedented insights into the impact of mismatches on the binding of over 20 TFs. For every tested TF, mismatches were identified that exerted either a positive or negative impact on binding [12]. This investigation demonstrates that mismatches can induce DNA distortions similar to those caused by TFs, effectively pre-paying the energetic cost associated with DNA conformational rearrangement and enhancing TF binding affinity. For example, whilst the introduction of a C-C mismatch in the TBP DNA binding site markedly improves binding affinity, crystallographic analysis demonstrates that the TBP/DNA contacts are similar in the bound complexes of TBP with wild-type and mismatched DNA binding sites (Figure 7). This suggests the mismatch improves binding by pre-bending the DNA into the active-binding conformation rather than by lowering the energy of the bound complex. This approach could now be extended to include additional damages and TFs, facilitating the exploration of conformational penalties in critical-binding reactions. With the advance of deep learning and large-scale high-throughput experimental data, computational models can now predict the binding of proteins to binding sites containing mismatches. For example, DeepRec can predict relative-binding affinities as a function of physicochemical signatures and the effect of DNA methylation or other chemical modifications on binding [128].

Overall, the capacity of DNA mismatches to modify DNA conformation and perturb the free energy changes of binding reactions further highlights the critical role of DNA shape in determining recognition by TFs. This suggests a nuanced interplay between DNA structural alterations and the regulatory dynamics of gene expression.

### Bulky adducts

**Figure 8.**
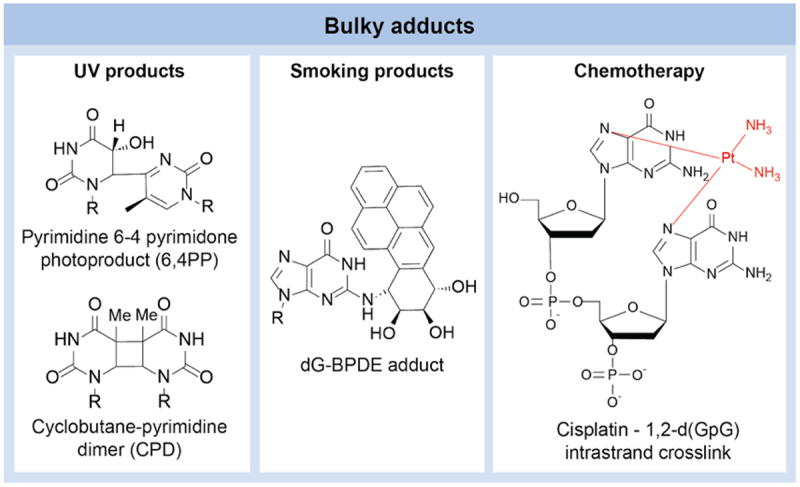
Different types of bulky adducts formed by DNA damage agents. Left panel: UV-induced formation of pyrimidine/pyrimidones and cyclobutane dimers. Central panel: product of BaP diol-epoxide addition to guanine. Right: intra-strand crosslinking between neighboring guanine residues by cisplatin.

The three types of DNA damage discussed above are characterized by their small size and confinement to a single base pair or specific region of the DNA backbone. However, another particularly harmful category of DNA damages are the bulky adducts that distort the helical structure of DNA, posing significant obstacles to transcription and replication processes. Representative examples of bulky adducts are depicted in Figure 8. These adducts can arise from a variety of sources and significantly alter DNA structure. For example, they can be formed by the covalent binding of chemical carcinogens with large sizes, such as polyaromatic hydrocarbons (PAHs), aromatic amines, and the chemotherapy reagent cisplatin, to various sites on DNA bases [129]. Alternatively, UV light can provide the energy for excited state photochemistry that allows reactivity between DNA bases themselves. The presence of bulky adducts on DNA can profoundly affect the recognition capabilities of TFs, illustrating the diverse mechanisms through which DNA integrity can be compromised and the complex challenges posed to cellular regulatory systems. In this section we briefly survey these different adduct classes and consider their potential impact on DNA-TF recognition.

Benzo[a]pyrene (BaP) and other polycyclic aromatic hydrocarbons (PAHs), found in cigarette smoke, are prevalent contaminants in air and food with genotoxic properties, leading to DNA damage [130]. Covalent interaction of the carcinogenic compound BaP diol-epoxide (BPDE) with DNA induces conformational changes in the DNA in the vicinity of the adduct formation, leading to an influence on TF binding [131,132]. The interaction of the TF Sp1 with GC-box sequences in the promoter of the hamster adenosine phosphoribosyl transferase gene serves as a valuable model system. Surprisingly, when this DNA fragment was highly modified with BPDE, there was a considerable increase (ranging from 5 to 10 times) in apparent binding affinity for Sp1 [131]. Further investigation has revealed that BPDE can induce DNA bending even in sites lacking the normal GC-box Sp1 binding sites. This induced bending mimics the conformation of the normal GC box-Sp1 complex, consequently enhancing Sp1 binding even in sites devoid of the non-GC box [133]. Similar results were shown for E2F, while for GAL4 this damage did not result in enhanced affinity [134,135]. These findings suggest that TFs capable of bending DNA may exhibit anomalously high affinity for sequences containing carcinogen-DNA adducts, as the energetic penalty of complex formation is offset by the preexisting DNA distortion.

Formation of bulky adducts on DNA bases is also the mechanism of action of several chemotherapy drugs. Since its clinical introduction in 1978, cisplatin [cis-diamminedichloroplatinum(II)] has been established as a primary drug for treating lung, colorectal, ovarian, and head-and-neck cancers in chemotherapy regimens [136]. Its effectiveness lies in its ability to damage cancer cell DNA, predominantly by creating Pt-d(GpG) intrastrand diadducts and, to a lesser extent, Pt-d(ApG) and Pt-d(GpXpG) intrastrand diadducts and Pt-G-G interstrand cross-links [137–139]. These damages have been mapped in the human genome at single nucleotide resolution [140], and gene architecture was identified as a major determinant of the transcriptional response that is hardwired into the human genome [141]. Research has demonstrated that the promoter recognition factor TBP selectively binds to DNA damaged by cisplatin, whether in isolation or within a larger protein complex like TFIIH [142]. This affinity is partly explained by the structural similarity, as revealed by 3D analysis, between the TATA box in the TBP complex and DNA altered by cisplatin, suggesting that such damaged DNA might compete for TBP/TFIID binding, thereby contributing to DNA damage-induced inhibition of RNA synthesis. These conclusions agree with the suggested mechanism for other bulky adducts described above, further highlighting the importance of damaged DNA in cellular processes.

Two additional types of bulky DNA damage, known as cyclobutane pyrimidine dimers (CPDs) and pyrimidine (6–4) pyrimidone photoproducts (64PPs), are frequently formed as a result of exposure to UV light. In recent years, technological advancements and the development of new assays have provided systematic insights into the interplay between UV damage and protein-DNA recognition. For instance, a study characterized the sequence dependency of UV damage by probing a complete pool of 4096 hexamer sequences using high-throughput sequencing [143]. Additionally, the high-throughput chip-based assay UV Bind revealed significant alterations in TF binding specificity following UV exposure [144]. The interplay between TF binding and its potential impact on damage formation, repair, and mutagenesis has been the subject of multiple studies in recent years [37,85,145–149].

Beyond the damages considered above, many others exist in the human genome resulting from metabolic or hydrolytic processes and external sources. Several additional examples have demonstrated the impact of bulky adducts on TF binding [150,151] Other damages, such as alkylated DNA products O2- and O4-alkylated thymidine lesions, were shown to be bound by three high-mobility group (HMG) proteins (HMGB1, HMGB2, and mitochondrial transcription factor A (TFAM)) preferentially (33290046). These additional damages may also have a significant impact on TF-DNA binding. The discoveries above underscore the necessity to further explore and advance research in this field in order to get a better picture of how various cellular processes are affected by the interplay between DNA damage and TF-DNA recognition.

In conclusion, bulky DNA adducts significantly influence TF recognition. Due to their large size and structural complexity, these adducts can induce substantial alterations in DNA conformation, thereby hindering the binding affinity and specificity of TFs, while helping others. This perturbation of TF-DNA interactions disrupts normal gene regulation, underlining the critical role of DNA integrity in maintaining cellular function and genomic stability. Further research into the mechanisms underlying the effects of bulky adducts on TF recognition could offer valuable insights for the development of targeted therapeutic strategies for diseases associated with DNA damage and mutation. Exciting advancements, including new genomic characterization methods [140,152–155], high-throughput assays for in vitro characterizations, and single-molecule assays for following DNA-binding kinetics of proteins from nuclear extracts (SMADNE) to damaged DNA [156], hold promise for significant progress in this field in the near future.

## DNA mechanical stress

Beyond alterations to the chemical structure of DNA which perturb both direct and indirect sequences readout by TFs, recognition of DNA by proteins may also be perturbed by structural changes that occur due mechanical perturbations of the DNA structure in the cellular environment. For instance, cellular DNA packaging and specific DNA-binding proteins can induce bent DNA structures, while mechanical strain from transcribing and replicating polymerases can produce supercoiled configurations such as underwound DNA. These modifications expand the topological repertoire of DNA beyond the canonical B-form. In this section, we consider evidence for the role such mechanical deformations play in TF-DNA recognition.

### DNA bending

TFs binding often induces local deformations in DNA structure, such as bending along its helical axis [157]. This bending process has been suggested to play a role in regulating transcription through various mechanisms. For example, bending may alter direct contacts between functional groups in the protein and DNA [158,159], or pre-organize the DNA into its preferred binding conformation, partially offsetting the energetic penalty associated with forming the transition state of the binding reaction. One of the most well-known examples of a DNA bending protein is the TATA-binding protein (TBP), a crucial component of the transcription initiation complex in eukaryotes. TBP facilitates the recruitment of RNA polymerase and other TFs. The crystal structures of multiple TBP-DNA complexes reveal that TBP binds to the minor groove of the TATA box, unwinding the TATA domain by ~105 degrees and inducing bending of the DNA toward the major groove by ~80 degrees, significantly deforming the DNA [24,160–162]. Molecular dynamic simulations show that inherent DNA curvature and flexibility correlate with TATA box functionality [163] and a connection between DNA bending and the affinity of TBP has been established [164]. Indeed, pre-bending of a promoter sequence can increase TBP binding affinity by 100-fold [165]. It is proposed that the requirement for DNA deformation contributes to the comparatively slow rate of TBP binding [166]. The Estrogen Receptor also binds DNA in a bent form [167] and replacing a single consensus Estrogen Response Element (ERE), which undergoes a 56-degree bend upon binding to Estrogen Receptor (ER), with an intrinsic DNA bending sequence of 54 degrees effectively activated transcription [167]. Many other examples of TFs binding DNA in a highly bent conformation include LEF-1, SRY, and YY1 [167–173], while more subtle bending also occurs in numerous other TFs [12]. While these examples illustrate the influence of DNA bendability on TF-DNA interactions, the complete impact of bending on most TFs remains unknown. Beyond direct TF interaction, DNA bendability significantly influences nucleosome positioning, a major determinant of genomic TF binding [174]. Therefore, DNA pre-bending or bending flexibility may exhibit secondary effects on TF binding activity, with interactions between different TFs upon bending further contributing to these secondary effects. Furthermore, the interplay between modifications and DNA bendability, as evidenced by recent observations demonstrating the impact of CpG methylation on DNA flexibility [52], adds another layer of complexity. Despite advancements in genomic methods and in vitro binding assays, our comprehension of these phenomena remains incomplete, and a significant effort will be required to fully capture its effect.

### DNA supercoiling

Torsional forces (the forces applied in twisting an elongated object) arise during fundamental processes such as replication, DNA repair and transcription [175,176]. These deformations result in supercoiling (underwinding or overwinding the DNA), local DNA melting, base flipping, twisting, and the formation of additional non-canonical structures such as loops, cruciforms and left-handed Z-DNA [177–179].

While supercoiled structures have fascinated the scientific community for several decades [180–182]. One of the well-studied TFs in the context of supercoiling and other DNA distortions is the Integration Host Factor (IHF), a bacterial protein with a pivotal role in regulating DNA structure and gene expression. IHF induces one of the sharpest bends observed in DNA, with an estimated angle of approximately 160 degrees [183]. The curvature of supercoiled DNA, which is heightened, facilitates its wrapping around IHF. Conversely, IHF acts as a “supercoiling relief” factor, compacting relaxed DNA loops and specifically positioning plectonemes in supercoiled DNA. Moreover, IHF regulates under- or overtwisted DNA, acting as a “supercoiling buffer” regardless of whether the complex forms in negatively or positively supercoiled DNA [184]. A similar mechanism has been suggested for TBP. The hypothesized mechanism for diminishing TBP-induced writhe indicates that TBP might cause a change in twisting (ΔTw) from 0 to −0.3 in minicircles by reducing out-of-plane bending when retracting its intercalating Phe stirrups, effectively acting as a “supercoil shock absorber” [185].

**Figure 9.**
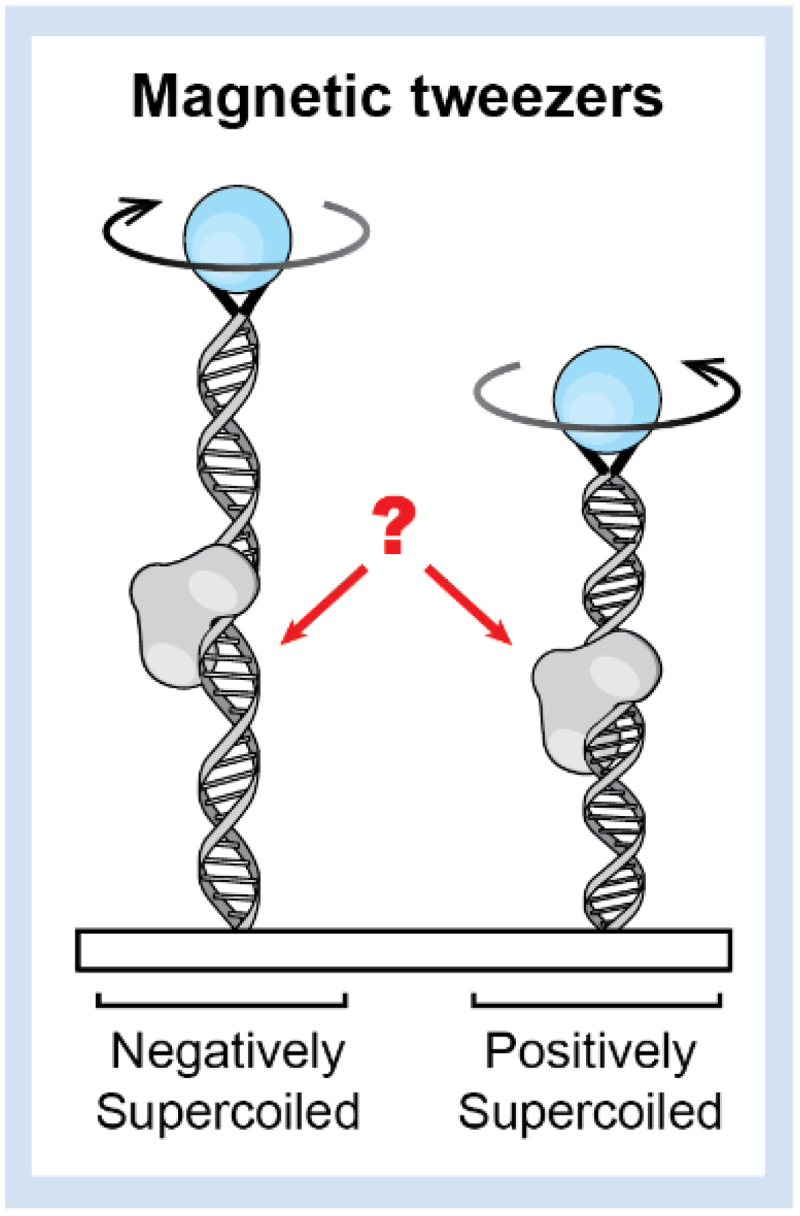
Schematic representation of introduction of negative and positive supercoiling in TF-binding DNA by magnetic tweezers.

Recently, novel methods have been developed to create positively or negatively supercoiled DNA. For instance, Optical DNA Supercoiling (ODS) is a single-molecule method based on dual-trap optical tweezers that permits the controlled formation and study of supercoiled DNA [186]. ODS also enables the imaging of supercoiled DNA with fluorescence microscopy and the direct visualization and quantification of protein dynamics on supercoiled DNA. A study utilizing ODS demonstrated that the diffusion of the mitochondrial transcription factor TFAM is significantly hindered by local regions of underwound DNA [186]. Multiplex flow magnetic tweezers were also utilized to generate supercoiled DNA and detect rare events in the cell cycle (Figure 9). This method characterizes the supercoiling dynamics and drug-induced DNA break intermediates of topoisomerases with single-molecule precision [187]. A particularly interesting example is the observed influence of DNA supercoiling on the activity of the CRISPR-Cas9 system, extensively utilized in the past decade as a genome-editing tool. Single-molecule optical tweezers experiments have revealed that negatively supercoiled DNA induces off-target binding of Cas9, which can lead to unwanted and potentially harmful reactions in the genome. With CIRCLE-seq, over 10,000 off-target double-strand breaks caused by negative supercoiling were identified across the genome. These results were also validated in vivo, demonstrating that Cas9 off-target activity could be induced by cellular processes that alter DNA topology, such as transcription and replication [188]. While the CRISPR-Cas9 system does not directly involve transcription factors, this example nevertheless serves to illustrate the relationship between DNA torsional stress and protein binding, suggesting further roles for supercoiling in regulating DNA-TF recognition in vivo. Moreover, recent advances in genome-wide mapping technologies have revealed that supercoiled structures may extend more than 1000 bp from a transcribing polymerase [189,190]. In light of these observations, further investigation into the impact of supercoiling is necessary to fully understand the role it plays in regulating TF binding.

In conclusion, significant evidence suggests bent and supercoiled DNA may play a key role in regulating TF binding, underscoring a fundamental aspect of the transcriptional machinery’s operation and emphasizing the nuanced interplay between DNA architecture and protein–DNA interactions. Bending can prepay the energetic costs of TF binding, whilst supercoiling can profoundly affect the availability of binding sites to TFs, thereby modulating their binding efficiency and fidelity. This relationship suggests a sophisticated level of regulation that goes beyond simple DNA sequence recognition into the realm of structural dynamics. However, the detailed mechanisms through which mechanical stress influences TF binding are not fully understood and require further investigation. By focusing research efforts on this area, we can anticipate gaining deeper insights into the molecular basis of TF binding specificity and affinity, with implications for understanding cellular processes and developing new therapeutic approaches.

Beyond the mechanical perturbations discussed above, many other DNA deformations and non-canonical secondary structures can form in the human genome, for example, G-quadruplexes, i-motifscruciform structures, Holliday junctions, and more. These structures might also present binding sites for TFs with unique affinity and selectivity profiles. Whilst a detailed review of these alternative DNA structures is beyond the scope of the present review, they nonetheless provide an interesting avenue to explore in the quest to understand the dynamic nature of cellular DNA on TF recognition.

## Protein post-translational modifications (PTMs)

In addition to the dynamic changes that occur in DNA, the role of modifications to the structure of the respective TF binding partner must also be considered. Indeed, proteins also undergo dynamic alterations that have the potential to impact TF-DNA recognition in the cell. These include structural changes, particularly in the dynamic intrinsically disordered domain of TFs. Indeed, the contribution of such effects to binding specificity in primary cells is becoming ever clearer [191–194]. Additionally, chemical changes, such as post-translational modifications (PTMs) are the subject of extensive research, but their impact on the various mechanisms of DNA recognition in the genome is far from fully understood [195–198].

**Figure 10.**
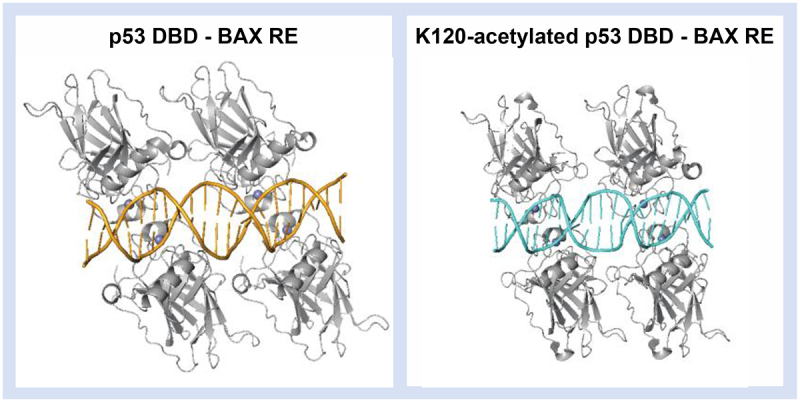
Crystal structure of BAX response element bound to (left) p53 DNA binding domain (PDB: 4HJE) and (right) K120-acetylated p53 DNA binding domain (PDB: 5LGY). The introduction of the PTM requires the DNA to adopt a different conformation in the bound complex.

PTMs are dynamic alterations that modulate protein structure and function, playing a vital regulatory role in proteome diversity [199]. Hundreds of PTMs have been discovered, distributed across various sites and residues, including within the polypeptide backbone, specific amino acid residues, and at *N*- and C-termini [200]. TFs are known to undergo a variety of PTMs at multiple positions and combinations, such as phosphorylation, acetylation, glycosylation, and ubiquitination [201]. These modifications can regulate TF stability, protein – protein/protein–DNA interactions, and subcellular localizations [202–204]. In particular, PTMs in the TF binding domain can directly influence the residues interacting with DNA, shaping “direct readout.” Additionally, these modifications may impact “indirect readout,” altering their preferences for certain DNA structures. For example, crystallographic analysis showed that acetylation of p53 at K120 requires a different conformation of the BAX response element in the bound TF-DNA complex (Figure 10). Another significant effect of PTMs may arise when these modifications alter TF interactions with other proteins, including the crucial partnering of TFs and the nucleosome. Naturally, if PTMs affect TF stability or cellular localization, they can also influence the overall TF concentration in the nucleus and, consequently, their overall binding occupancy. Consequently, such natural transformations have a critical impact on the recognition and binding events between TFs and DNA. Therefore, while accurate and controlled PTMs in TFs are essential for maintaining the proper functionality of gene expression programs in living cells, misregulation of PTMs can lead to aberrant gene expression [205–207].

Although predicting the structural impact of some modifications is possible, unraveling the molecular roles of most PTMs on TFs remains a challenge. One obstacle in gaining insights into the specific effects of defined PTMs at certain sites is the difficulty of obtaining homogeneously modified TF analogs. Achieving a desired modification pattern at selected sites proves challenging due to limitations in traditional biological tools, such as cellular expression, mutagenesis, and enzymatic approaches. Other biological methods, such as genetic code expansion technology, allow for the effective incorporation of PTMs into proteins via engineered orthogonal aminoacyl-tRNA synthetase/tRNA pairs [208,209]. However, this method relies on the availability of an orthogonal tRNA synthetase for the target PTM, and it is challenging to apply for installing multiple PTMs and/or at different sites [210]. Recent advancements in synthetic tools offer a promising strategy for facing this challenge, providing an unprecedented opportunity to prepare defined, site-specifically modified analogs with atomic resolution [211–213]. Solid-phase peptide synthesis enables the precise insertion of virtually any desired PTM during peptide elongation on the solid support. Subsequently, the modified synthetic polypeptides are assembled in solution using chemoselective ligation approaches to yield the desired full-length modified proteins [214–216]. Alternatively, recent developments in late-stage functionalization chemistry allow unprecedented opportunities to transfer novel PTM mimicry into recombinant proteins [211], mainly by chemoselective modification of engineered reactive sites, e.g., cysteine residue through S-alkylation chemistry, Michael addition, or carbon–carbon bond formation reactions [213,217,218].

While there is still a long road ahead to understand the impact of all PTMs on some of the most important regulatory TFs, we envision that the integration of advanced high-throughput technologies with new synthetic approaches will pave the way for producing novel modified TFs and studying their binding preferences to DNA [219–221]. This, in turn, will aid in decoding the full impact of PTMs on gene expression regulation in both health and disease [220,221].

## Discussion

The fundamental event of a TF binding to DNA is primarily determined by the interactions between the two entities and the overall free energy gained in forming the TF-DNA complex. However, within the genome, numerous additional factors can alter these interactions and subsequently influence the complex gene regulatory network within the cell. Some of these factors, identified in the present review, arise from dynamic changes continually occurring in the genome and the TF proteins themselves. These changes may be intentional and controlled, such as DNA epigenetic modifications and post-translational modifications, or they may result from cellular activities and reactions, such as DNA damage and the formation of non-canonical DNA structures.

The previous sections identify a substantial body of evidence that demonstrates that these dynamic changes can lead to alterations in TF binding, gene expression, and DNA repair. However, in each case, knowledge is currently limited to only a few TF proteins and DNA structures. Whilst these examples are illuminating, obtaining a comprehensive genome-wide overview of how these dynamic factors govern DNA-TF regulation is of vital importance to fully understanding the role these processes play in determining gene regulation in health and disease. Toward this end, more systematic studies probing the effect of modifications on TF-DNA recognition at the genomic scale would be welcome. Whilst this endeavor is non-trivial, recent developments pave the way to undertake such efforts, particularly the continuous development of high throughput methodologies for exhaustively mapping TF binding preferences to thousands of sequence contexts containing both canonical and non-canonical DNA bases. Recent studies have highlighted the potential of these techniques to exhaustively map TF binding preferences to mismatched and UV-lesioned DNA, acting as a stepping stone to deciphering the dynamic code of these modifications across the genome. We anticipate that future efforts to apply these techniques toward the study of other modifications will lead to additional insights that allow us to establish the role of these modifications in influencing TF recognition.

Understanding the impact of DNA mechanical stresses also remains a significant challenge. Whilst several platforms to induce strain into DNA have been introduced and used as models to probe these effects on protein binding in selected contexts, adapting them for use in high throughput experiments necessary to understand the effect of mechanical stress across the full range of TFs and canonical/modified DNA sequence contexts is, hitherto, unrealized. Beyond high throughput approaches that deepen our understanding of TF-DNA recognition at the genome level, recent developments in single-molecule assays (e.g., optical tweezers) offer enhanced capabilities for dissecting the mechanistic underpinnings of structural and mechanical modifications on TF-DNA recognition. This fundamental molecular understanding will not only deepen our knowledge of how these dynamic changes affect TF-DNA recognition, but may eventually allow the rational design of artificial TF-binding oligonucleotides with enhanced properties (e.g., affinity, and biostability) compared to their native counterparts for use in diagnostic or therapeutic applications. As such, ongoing advancements in experimental techniques hold promise for deepening our understanding of how chemical and structural modifications shape gene regulatory networks and cellular processes.
